# CCR7 enhances TGF-β1-induced epithelial-mesenchymal transition and is associated with lymph node metastasis and poor overall survival in gastric cancer

**DOI:** 10.18632/oncotarget.4484

**Published:** 2015-07-06

**Authors:** Huiying Ma, Lingling Gao, Shichao Li, Jie Qin, Long Chen, Xinzhou Liu, Pingping Xu, Fei Wang, Honglei Xiao, Shuang Zhou, Qiang Gao, Binbin Liu, Yihong Sun, Chunmin Liang

**Affiliations:** ^1^ Lab of Tumor Immunology, Department of Anatomy and Histology & Embryology, Shanghai Medical College of Fudan University, Shanghai, P. R. China; ^2^ The General Surgery Department of Zhongshan Hospital, Shanghai Medical College of Fudan University, Shanghai, P. R. China; ^3^ The Liver Cancer Institute of Zhongshan Hospital, Shanghai Medical College of Fudan University, Shanghai, P. R. China; ^4^ The Department of Anatomy and Histology & Embryology, Tongji University School of Medicine, Shanghai, P. R. China

**Keywords:** gastric cancer, CCR7, epithelial-mesenchymal transition, TGF-β1, co-stimulatory

## Abstract

CCR7 is a G protein-coupled chemokine receptor. In this study, we used immunohistochemistry with tissue microarrays to measure CCR7 expression in tumor specimens from 122 patients with gastric cancer. We show that CCR7 expression is associated with lymph node metastasis (*P* = 0.022) and overall survival (OS; *P* = 0.025), and is an independent factor associated with poorer overall survival (*P* = 0.032). The CCR7 mechanism was predicted based on bioinformatic analysis and verified in gastric cancer cell lines and primary tumor samples. The data show that CCR7 contributes to TGF-β1-induced epithelial-mesenchymal transition (EMT) and that the effects of TGF-β1 are inhibited by a CCR7 neutralizing antibody or a NF-κB inhibitor. Increased TGF-β1 expression was accompanied by nuclear localization of NF-κB-p65 and higher levels of the mesenchymal marker vimentin in human gastric cancer samples. We conclude that the CCR7 axis mediates TGF-β1-induced EMT via crosstalk with NF-κB signaling, facilitating lymph node metastasis and poorer overall survival in patients with gastric cancer. These findings suggest CCR7 is a novel prognostic indicator and a potential target for gastric cancer therapy.

## INTRODUCTION

With nearly one million new cases annually worldwide, gastric cancer (GC) is the fifth most common malignant disease and the second leading cause of cancer mortality. Despite improvements in diagnostic and treatment strategies, overall survival (OS) among GC patients remains poor, owing to high incidences of metastasis and recurrence. Chemokines and their receptors are key mediators of tumor invasion and metastasis [1–4]. Of particular interest to us is the chemokine receptor CCR7, a G protein-coupled seven transmembrane domain receptor with two high-affinity ligands, secondary lymphoid chemokine (SLC, also known as CCL21) and EB11-ligand chemokine (ELC, also known as CCL19). During normal immune responses, CCR7 initiates adaptive immune responses by mediating T cell and dendritic cell migration to lymph nodes [5]. In similar fashion, however, CCR7 expression by tumor cells increases the likelihood of lymphatic invasion and lymph node metastasis and correlates with metastasis in breast cancer, squamous cell carcinoma of the head and neck, and colon cancer [6–9]. The prognostic value of CCR7 expression has also been demonstrated in gastric cancer [10, 11]. We previously showed high CXCR6 expression to be an independent prognostic factor associated with invasive growth accompanied by an influx of CD66b^+^ neutrophils and microvessel growth in human hepatocellular carcinoma [1]. We also found a significant association between CCR7 expression and poor OS and enhanced infiltration of Tregs into primary tumor sites in GC [12]. As yet, however, there is no direct evidence to explain how chemokine receptors mediate tumor cell invasion and metastasis.

Epithelial-mesenchymal transition (EMT) is a process by which epithelial cells acquire a mesenchymal cell phenotype. In this process, cells gradually lose the epithelial characteristic of cell-cell adhesion, thereby gaining increased motility and invasiveness, and become resistant to apoptosis. EMT is thus considered to be a key process promoting tumor metastasis in epithelial cancers [13–15]. EMT can be induced by components in the tumor microenvironment such as TGF-β1 [16–19]. Moreover, one recent study showed that crosstalk between CXCR4 and TGF-β induces EMT in liver tumors [17].

Whether CCR7 participates in a similar process in GC is unclear. To address that issue, we investigated the relationship between CCR7 expression and TGF-β1-induced EMT in GC. Our findings suggest CCR7 facilitates TGF-β1-induced EMT and is thus a contributor to lymph node metastasis and poor survival among GC patients.

## RESULTS

### High CCR7 expression is an independent prognostic factor for OS in GC and associates with lymph node metastasis

**Figure 1:**
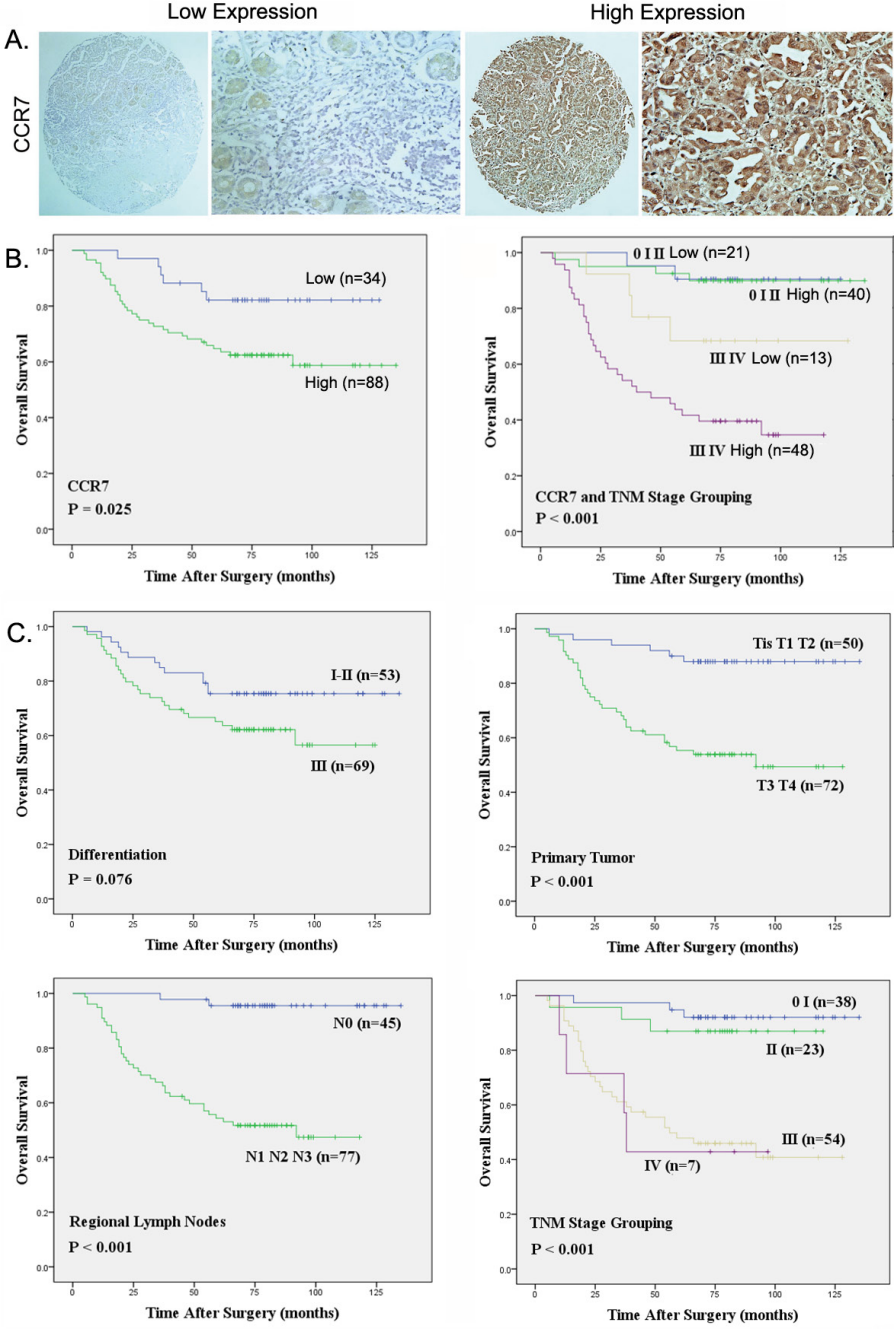
Strong CCR7 expression correlates with regional lymph node metastasis and poorer overall survival **A.** Representative images of cells expressing low or high levels of CCR7 in a primary gastric cancer tumor specimen (Magnification, ×400). **B.** Relation between overall survival and CCR7 expression (*P* = 0.025) and TNM stage (*P* < 0.001). **C.** Relation between overall survival and several clinical parameters, including differentiation (*P* = 0.076), primary tumor invasion (*P* < 0.001), regional lymph node involvement (*P* < 0.001) and TNM stage grouping (*P* < 0.001), respectively.

The clinical and pathologic characteristics of the 122 GC patients participating in the study are summarized in Table 1. CCR7 expression was analyzed immunohistochemically in microarrays of GC samples collected for this study. As shown in Figure 1A, CCR7 was diffusely distributed in both the cytoplasm and cell membrane of GC cells. CCR7 expression was strong in 36 (29%) samples, moderate in 52 (43%), weak in 21 (17%) and negative in 13 (11%). For further analysis, patients were divided into CCR7-low (negative and weak expression; *n* = 34) and CCR7-high (moderate and strong expression; *n* = 88) groups.

**Table 1:** Characteristics of the 122 gastric cancer patients

Variables	Number (%)
**Age (years)**	
< 60	63 (51.6%)
> = 60	59 (48.4%)
**Gender**	
Male	81 (66.4%)
Female	41 (33.6%)
**Tumor Size (cm)**	
**Median (mini-maximum)**	3 (1–11)
**Primary Tumor (T):**	
T1	29 (23.8%)
T2	21 (17.2%)
T3	70 (57.4%)
T4	2 (1.6%)
**Regional Lymph Nodes (N):**	
N0	45 (36.9%)
N1	51 (41.8%)
N2	20 (16.4%)
N3	6 (4.9%)
**Distant Metastasis (M):**	
M0	120 (98.4%)
M1	2 (1.6%)
**Stage Grouping (TNM):**	
Stage 0	4 (3.3%)
Stage IA–IB	34 (27.9%)
Stage II	23 (18.9%)
Stage IIIA–IIIB	54 (44.3%)
Stage IV	7 (5.3%)
**Differentiation:**	
I	9 (7.4%)
II	44 (36.1%)
III	69 (56.6%)

The correlation between the CCR7 expression level and clinical characteristics is summarized in Table 2. The data show that CCR7-high significantly correlates with regional lymph node metastasis (*P* = 0.022), whereas there is no significant correlation between the level of CCR7 expression with patients’ age, gender, tumor differentiation, primary tumor parameters, distant metastasis or TNM stage.

**Table 2:** Correlations between CCR7 and the clinical characteristics

Characteristics	CCR7		
	Low	High	*P*
**Age (years)**			
< 60	16	47	0.529
> = 60	18	41	
**Gender**			
Male	25	56	0.300
Female	9	32	
**LN Staging**			
N0	18	27	0.022*
N1–N3	16	61	
**Tumor Infiltration**			
T1–T2	15	35	0.662
T3–T4	19	53	
**TNM Staging**			
0–II	21	40	0.106
III–IV	13	48	
**Tumor Differentiation**			
I–II	18	35	0.188
III	16	53	

Univariate and multivariate analyses of the relation between CCR7 expression and the clinical characteristics are summarized in Table 3. CCR7-high was associated with poorer OS (log-rank test, *P* = 0.025), and CCR7 was an independent prognostic factor for OS (*P* = 0.032). When we then grouped the patients with respect to disease stage, the OS among patients in the CCR7-high group was similar to that among patients in CCR7-low group at stages 0, I and II (Figure 1B). On the other hand, patients in the CCR7-low group with stage III or IV disease lived significantly longer than those in the CCR7-high group (log-rank-test, *P* < 0.001). Moreover, TNM staging, lymph node metastasis and depth of infiltration were all associated with OS rates, whereas age, gender and tumor differentiation had no prognostic significance for OS (Figure 1C).

**Table 3:** Univariate and multivariate analysis of factors associated with OS

Variable	Univariate	Multivariate	
	*P*	HR	*P*
**Age (> 60 vs. < = 60)**	0.620	1.170	0.621
**Gender (female vs. male)**	0.144	0.630	0.149
**Differentiation**	0.076	0.554	0.081
**Regional lymph node**	< 0.001**	0.066	< 0.001**
**CCR7 (High vs. Low)**	0.025*	0.386	0.032*
**TNM stage (III-IV vs. 0-I-II)**	< 0.001**	0.127	< 0.001**

### High CCR7 expression increased mesenchymal-like phenotypes in GC cells and enhanced cell migration

**Figure 2:**
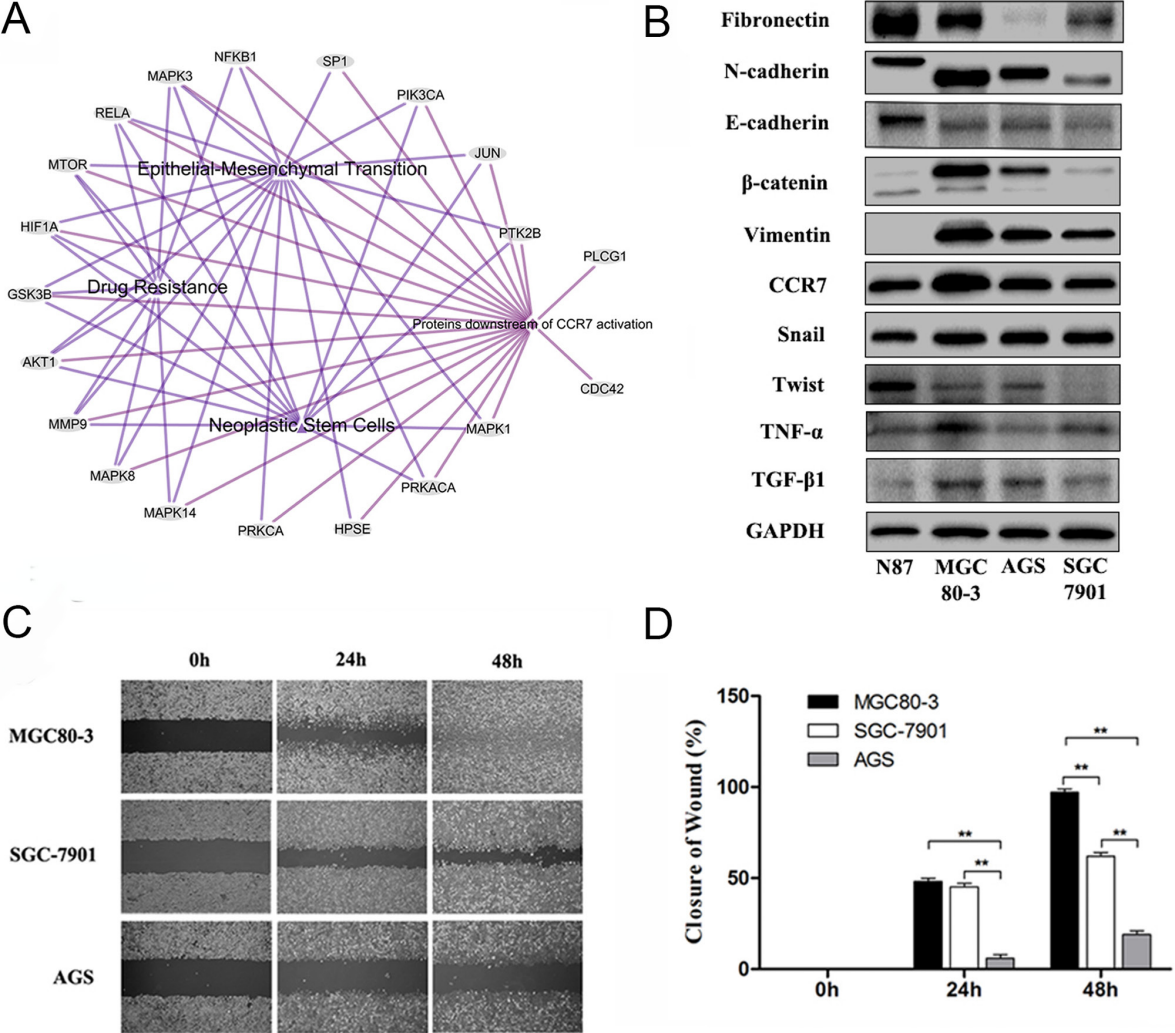
High CCR7 expression increases mesenchymal-like phenotypes in gastric cancer cells **A.** Bioinformatic analysis with 20 proteins involved in the CCR7 pathway sorted using EGAN and KEGG software. Among them, 18 proteins exhibited crosstalk with EMT, Drug Resistance and Tumor Stem Cell Signaling pathways. **B.** Expression of CCR7 and EMT-related proteins assessed by western blotting in the N87, MGC80-3, AGS, SGC-7901 gastric cancer cell lines. MGC80-3 cells showed greater expression of CCR7 as well as vimentin, β-catenin, N-cadherin and Snail. SGC7901 cells showed weaker CCR7 mesenchymal-related proteins. Higher levels of autocrine TGF-β1 and TNF-α were detected in MGC80-3 cells. **C.** and **D.** CCR7-high MGC80-3 cells exhibited a greater ability to migrate than CCR7-low SGC-7901 and AGS cells (magnification, 100×, **P < 0.01). Error bars depict means + SEM.

To further explore possible mechanisms related to EMT, we applied bioinformatic methods to predict the role of CCR7. Among 20 proteins in the CCR7 signaling pathway sorted using EGAN and KEGG software (Figure 2A), 18 were involved in crosstalk with EMT, drug resistance and tumor stem cell signal pathways. We therefore examined expression of CCR7 and EMT-related proteins in four human GC cell lines (Figure 2B). MGC80-3 cells expressed the highest levels of CCR7 as well as high levels of N-cadherin, β-catenin, vimentin and Snail, which is characteristic of a mesenchymal-like phenotype. SGC7901 cells showed weaker expression of both CCR7 and mesenchymal proteins. Notably, high levels of autocrine TGF-β1 and TNF-α were also detected in MGC80-3 cells. Consistent with their stronger CCR7 expression, MGC80-3 cells exhibited greater migration in wound-healing assays than CCR7-low SGC-7901 and AGS cells (Figure 2C, 2D).

### CCR7 is involved in TGF-β1-induced EMT *in vitro*

**Figure 3:**
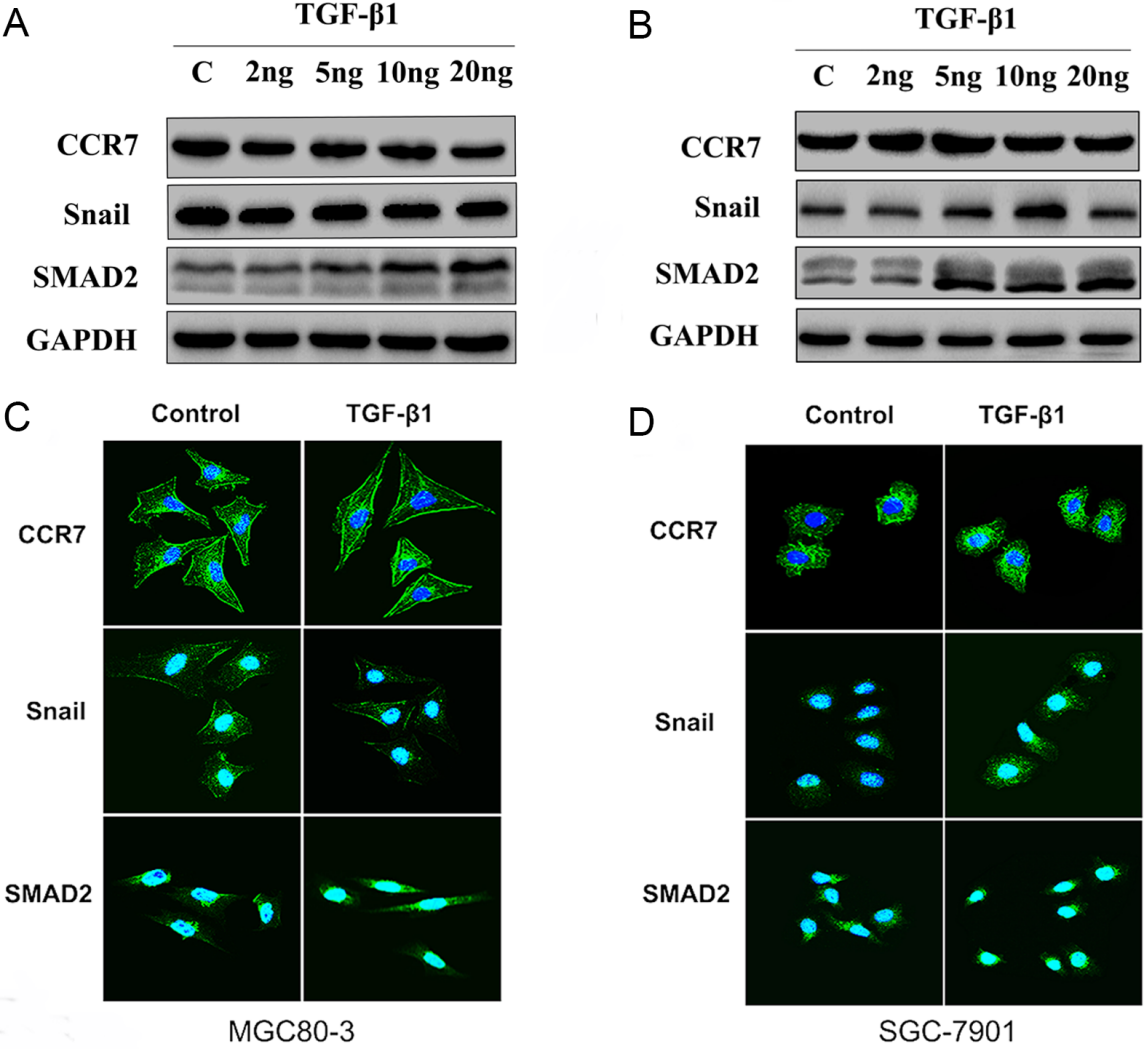
CCR7 contributes to TGF-β1-induced EMT *in vitro* **A.** and **C.** Western blotting and immunofluorescent staining of CCR7-high MGC80-3 cells revealed increased SMAD2 expression in cells treated with 10–20 ng/ml TGF-β1. There was no obvious alteration in Snail expression. **B.** and **D.** CCR7-low SGC-7901 cells showed increased expression of both SMAD2 and Snail when treated with 5 or 10 ng/ml TGF-β1. TGF-β1 treatment elicited no obvious effect on CCR7 expression in either cell line.

**Figure 4:**
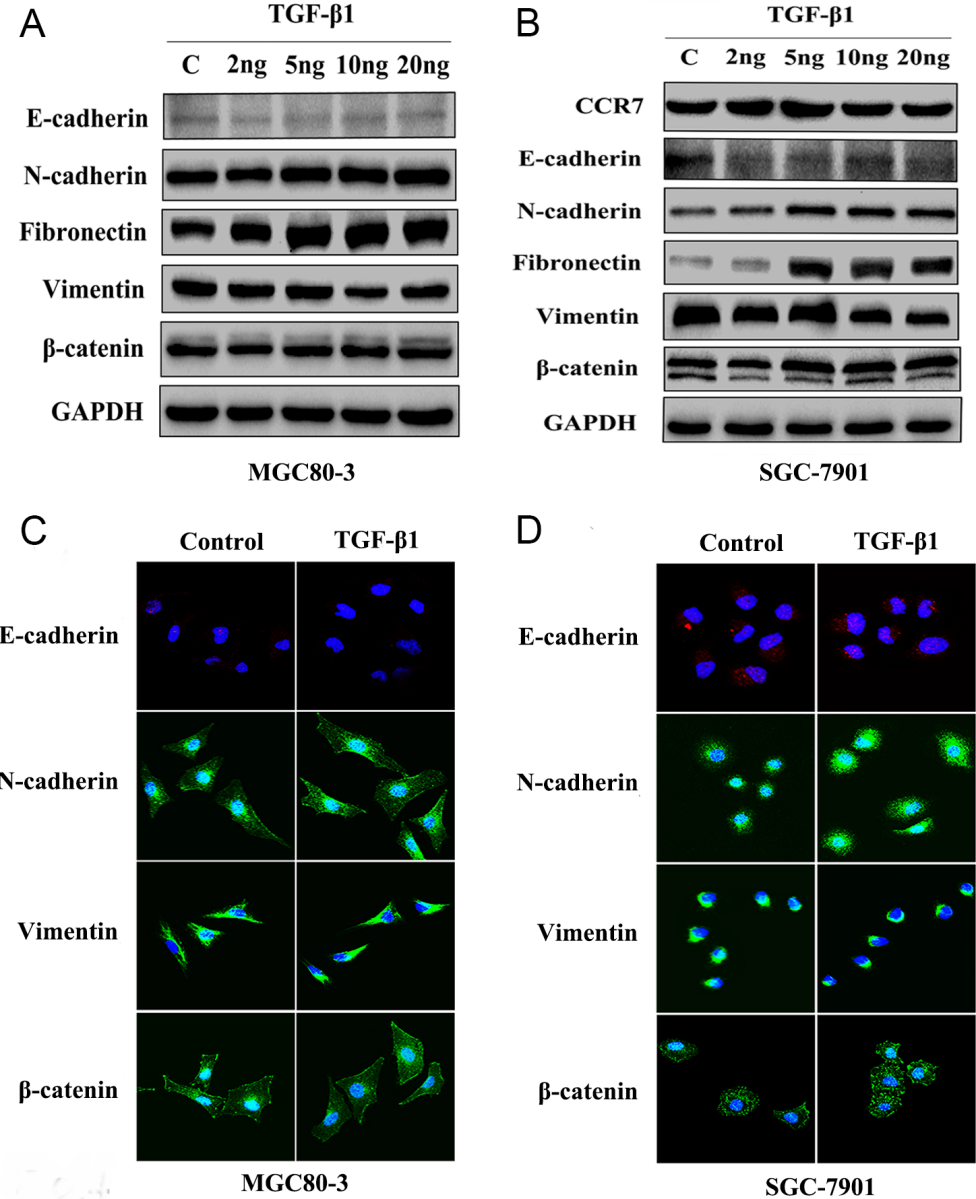
CCR7-low SGC-7901 cells were sensitive to TGF-β1-induced EMT *in vitro* **A.** and **C.** Western blotting and immunofluorescent staining of MGC80-3 cells revealed slightly increased expression of the mesenchymal-related proteins N-cadherin and fibronectin in cells treated with 5–20 ng/ml TGF-β1. There was no obvious alteration in β-catenin. **B.** and **D.** SGC-7901 cells showed greatly increased expression of N-cadherin, fibronectin and β-catenin when treated with 5–20 ng/ml TGF-β1. TGF-β1 treatment elicited no obvious effect on vimentin expression in either cell line.

EMT can reportedly be induced by various signals from the tumor microenvironment, including TGF-β1 [16, 20]. To test whether CCR7 is involved in TGF-β1-induced EMT, CCR7-high MGC80-3 cells and CCR7-low SGC-7901 cells were treated with selected concentrations of TGF-β1. As shown in Figure 3, SMAD2 was up-regulated in both cell lines, indicating the TGF-β1 signaling pathway was activated. This in turn led to alterations in the cells’ protein expression profile consistent with a mesenchymal phenotype. TGF-β1-treated MGC80-3 cells exhibited small increases in N-cadherin and fibronectin, while more pronounced increases were seen in SGC-7901 cells (Figure 4). This implies that CCR7-low tumor cells are more susceptible to TGF-β1-induced EMT.

### Involvement of CCR7 in TGF-β1-induced EMT and cross-talk with NF-κB

**Figure 5:**
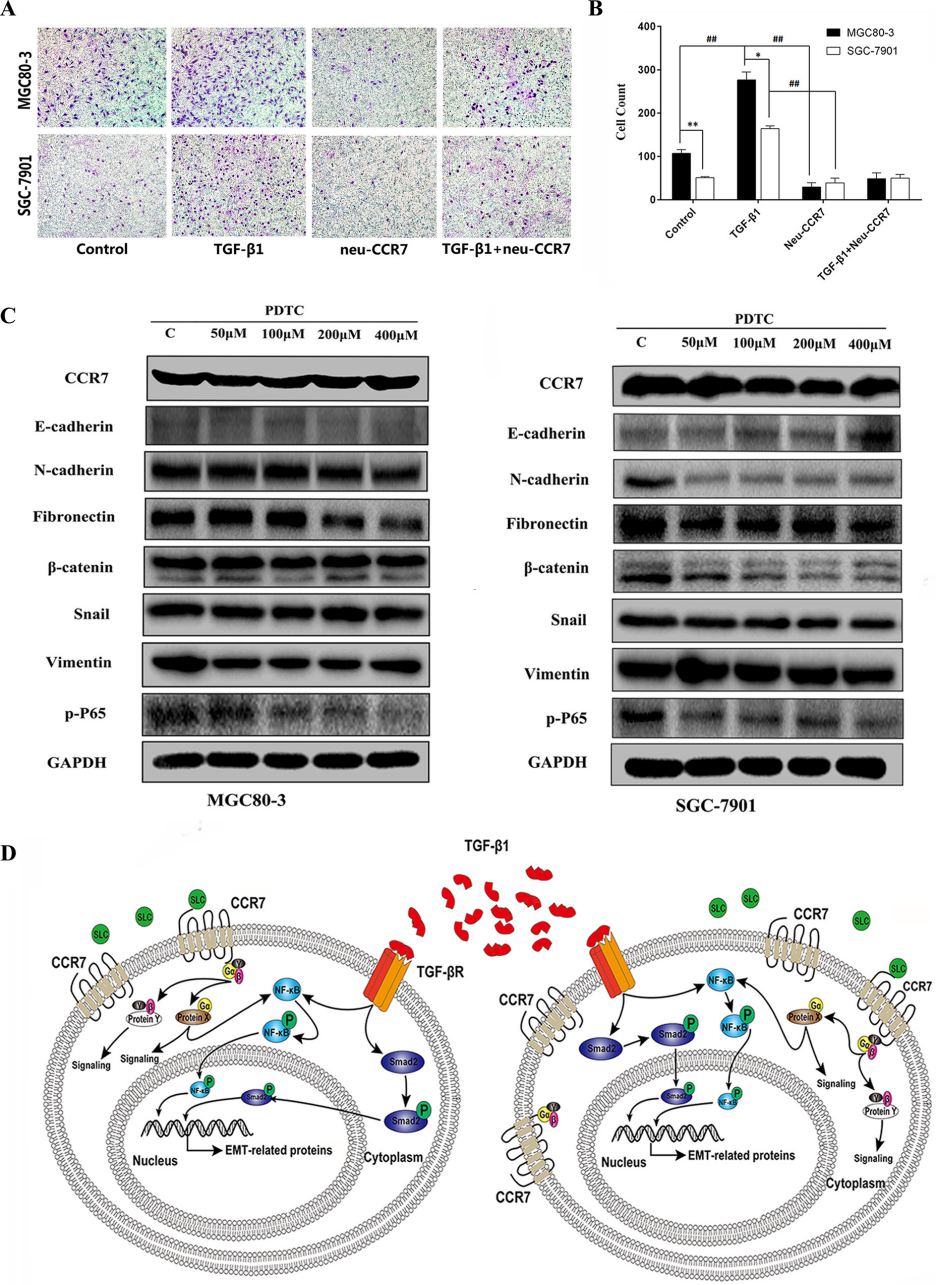
CCR7 and TGF-β1-induced EMT cross talked in NF-κB pathway **A.** and **B.** Transwell invasion assays showed that CCR7-high MGC80-3 cells are more invasive than CCR7-low SGC-7901 cells (**P < 0.01). TGF-β1 stimulation significantly increased the invasiveness of both tumor cell lines (^##^*P* < 0.01 vs. control), but the effect was totally blocked by neu-CCR7 antibody (^##^*P* < 0.01 vs. TGF-β1). **C.** In the presence of the indicated concentrations of PDTC, phosphorylated P65 levels were decreased and levels of the EMT-related proteins N-cadherin, fibronectin and β-catenin tended to be lower in both cell lines. **D.** Schema illustrating the proposed crosstalk between the SLC/CCR7 and TGF-β1/TGF-βR axes.

To further explore the contribution of CCR7 to the invasiveness of tumor cells, we next examined its effect on MGC80-3 and SGC-7901 cell migration in transwell invasion assays. As shown in Figure 5A, 5B, CCR7-high MGC80-3 cells were significantly (*P* < 0.01) more invasive than CCR7-low SGC-7901 cell. Moreover, TGF-β1 stimulation significantly (*P* < 0.01) increased the invasiveness of both tumor cell lines, and the effect was completely blocked by neu-CCR7, a neutralizing antibody, (*P* < 0.01). These results suggest there may be cross-talk between signaling in the SLC/CCR7 and TGF-β1/TGF-βR axes in GC cells, which affects their biological function.

The NF-κB pathway was recently reported to be situated downstream of the SLC/CCR7 axis in hepatocellular carcinoma and downstream of the TGF-β1/TGF-βR axis in prostate cancer [21, 22]. We therefore used pyrrolidine dithiocarbamate (PDTC), a NF-κB pathway inhibitor, to assess the role of NF-κB in the process of TGF-β1-induced EMT. As shown in Figure 5C, in the presence of different concentrations of PDTC, phosphorylated P65 levels were diminished in both cell lines. However, a higher concentration of PDTC was needed to inhibit the NF-κB pathway in CCR7-high MGC80-3 cells. Similarly, levels of most EMT-related proteins, including N-cadherin, fibronectin and β-catenin, tended to decline in the presence of PDTC. A schema showing the potential signal cross-talk between the SLC/CCR7 and TGF-β1/TGF-βR axes and NF-κB is summarized in Figure 5D.

### CCR7 facilitates EMT in clinical GC samples

**Figure 6:**
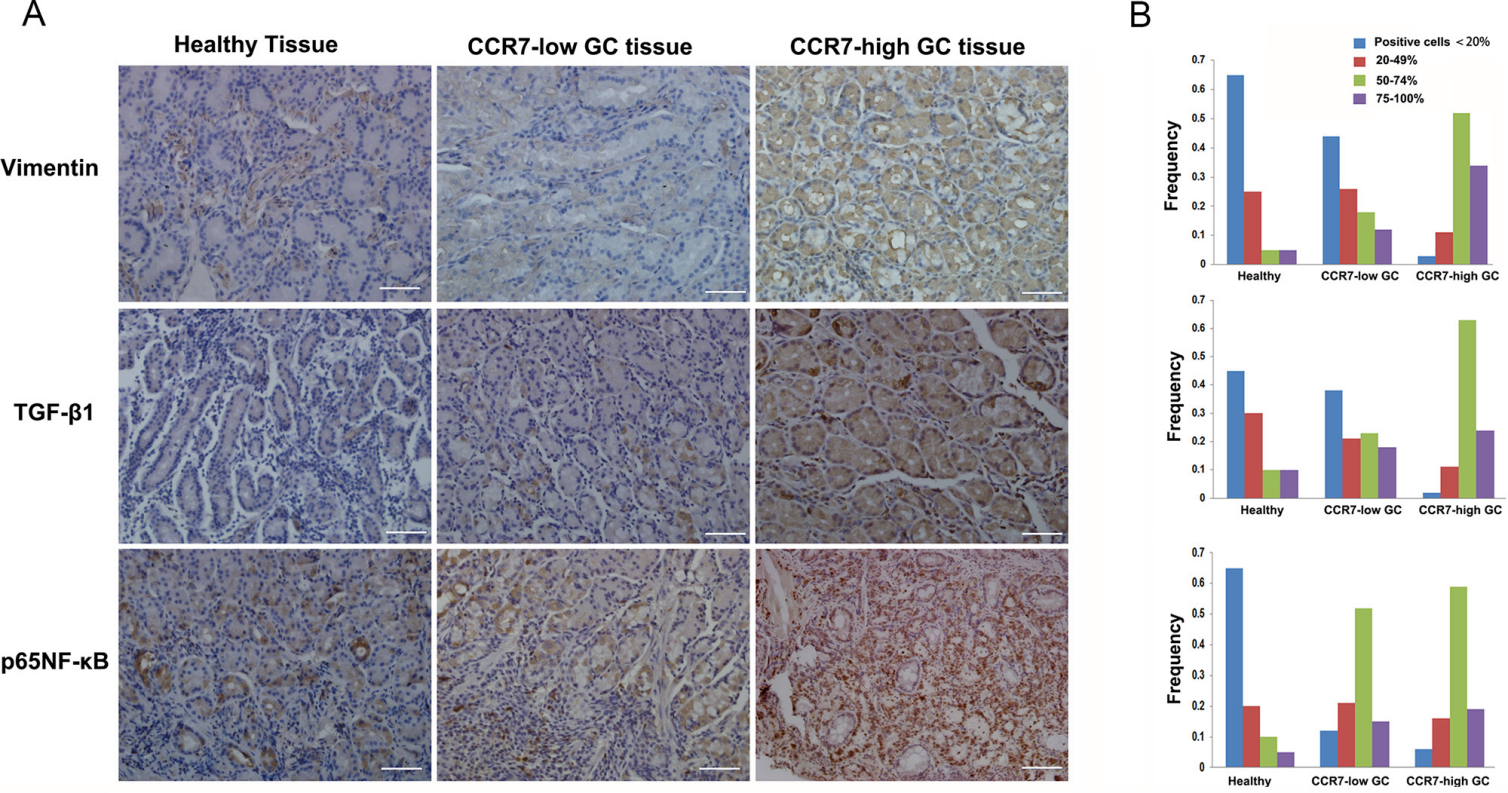
CCR7 facilitates EMT in clinical gastric cancer samples GC samples sorted into CCR7-high (*n* = 34) and CCR7-low (*n* = 88) groups and a set of healthy gastric samples (*n* = 20) were analyzed using immunohistochemistry. **A.** Immunostaining revealed stronger expression of vimentin and TGF-β1 in the cytoplasm of CCR7-high tumor cells. Increases in nuclear NF-κB-p65 were also detected (magnification, 200×). **B.** Corresponding frequencies of the four indicated staining levels were measured in healthy gastric samples, CCR7-low samples and CCR7-high samples. High levels of TGF-β1 expression (50–74% *plus* 75–100% positive cells) were detected in 87%, 41% and 10% of CCR7-high GC samples, CCR7-low GC samples and healthy gastric samples, respectively, while NF-κB-p65 was detected 78%, 67% and 15%, and vimentin was detected in 86%, 30% and 10%.

**Figure 7:**
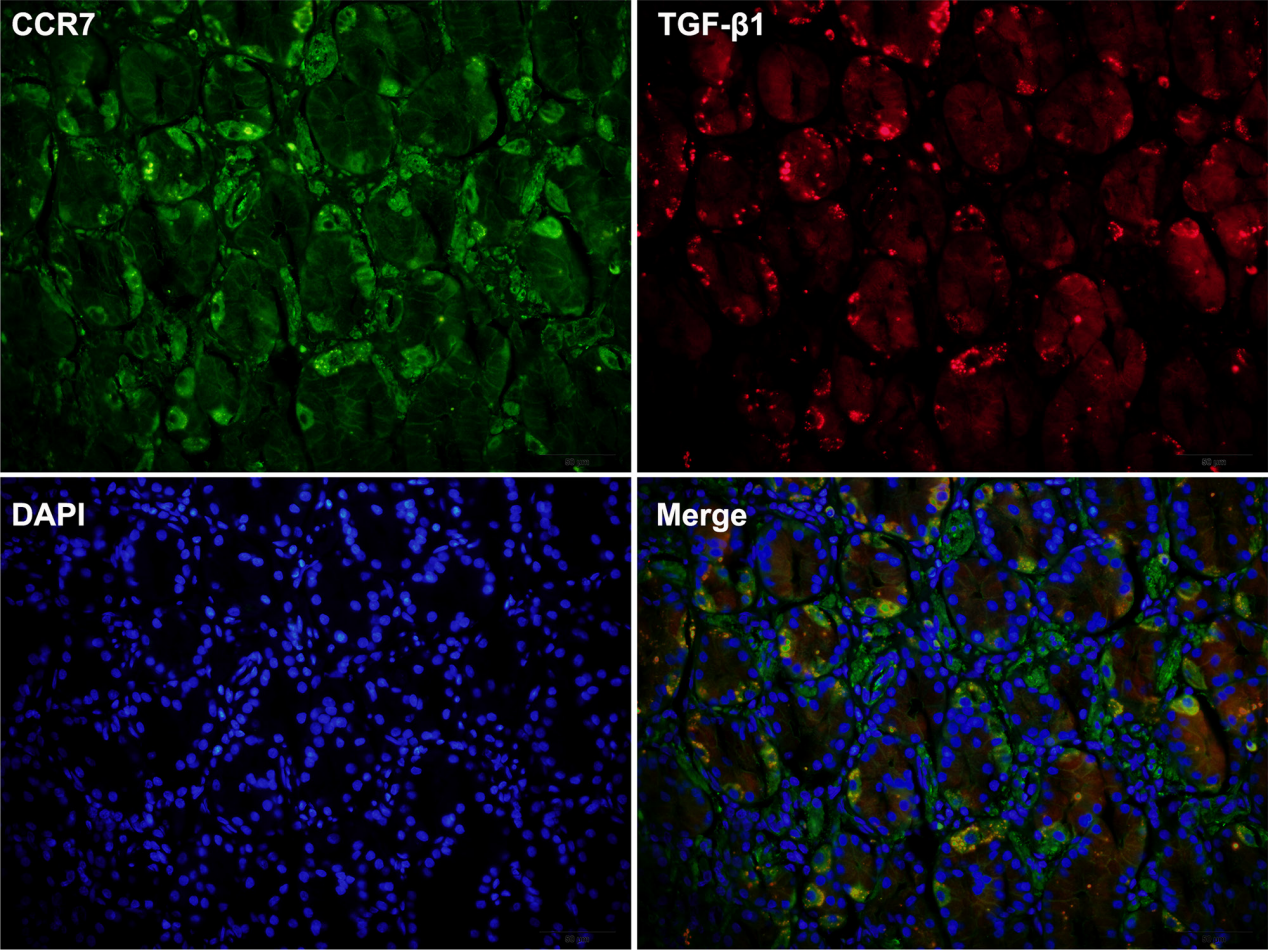
Double immunostaining revealed strong expression of TGF-β1 in CCR7-high gastric cancer samples Immunostaining CCR7-high GC samples (*n* = 34) revealed enhanced expression of TGF-β1 in the cytoplasm of tumor cells. The images shown are of serial sections from one representative patient (No. 25).

Finally, we used immunohistochemistry to compare CCR7-high and CCR7-low tumor samples from GC patients along with 20 samples of healthy gastric tissue, which served as a control. We found that increased TGF-β1 expression correlated with greater expression of the mesenchymal marker vimentin (Figure 6). Of note, a higher frequency of cells positive for nuclear pNF-κB-p65 was detected in CCR7-high samples, which further confirms activation of the NF-κB pathway. Double immunostaining also revealed higher levels of TGF-β1 in the cytoplasm of tumor cells in CCR7-high GC samples (Figure 7).

## DISCUSSION

We previously studied the association between CCR7 expression and GC patients followed up for 3 years [12]. Here, to explore the association between high and low CCR7 expression and clinic pathological factors and its utility as a prognostic indicator, we extended to follow-up to 5–10 years, making our results even more reliable. As seen earlier [6–9], Kaplan-Meier analyses revealed an association between TNM staging, lymph node metastasis, depth of infiltration and OS in GC. More interestingly, our multivariate analysis showed for the first time that high CCR7 expression is an independent prognostic factor for OS. Patients with stage III or IV GC and strong expression of CCR7 had worse OS than those weakly expressing the chemokine. On the other hand, CCR7 expression had no effect on OS among patients with stage 0, I or II GC.

Although high CCR7 expression has been implicated in tumor invasion and lymph node metastasis [10, 23–25], the associated mechanism and downstream mediators remain incompletely defined. In liver, lung and breast cancers, CCR7 functions with other molecules, including CCRL1, VEGF-C, COX-2 and microRNA let-7a, to regulate the metastatic activities of tumor cells [26–28]. To further explore the CCR7-related mechanism in tumor metastasis, we applied bioinformatic methods previously proven useful for identifying target genes or proteins [29, 30]. Using EGAN and KEGG software, we found that most proteins acting downstream of the CCR7 exhibited crosstalk with EMT. This finding prompted us to examine CCR7 expression and EMT-related proteins in GC cell lines. We found that MGC80-3 cells, which strongly express CCR7, also expressed high levels of autocrine TGF-β1, which likely served to maintain and/or enhance the mesenchymal cell phenotype. This was not the case with SGC-7901 GC cells, which expressed CCR7 only weakly. Unlike in earlier studies of lung and breast cancers [31–33], the results of the present study imply that GC cells may spontaneously acquire mesenchymal-like features induced by autocrine TGF-β1-driven constitutive activation of SMAD2, which is consistent with previous studies of human hepatocellular carcinoma cells [34, 35]. TGF-β1 is thus thought to be a key factor contributing to cancer progression, primarily via EMT-triggered metastasis [17, 22, 36–39]. By contrast, few studies have explored the relationship between TGF-β1 and chemokines in GC.

This is the first study exploring the effect of CCR7 on TGF-β1-induced EMT and coincides with a recent report demonstrating crosstalk between CXCR4 and TGF-β-induced EMT in hepatocellular carcinoma [17]. Our findings suggest CCR7 acts as a co-stimulator in TGF-β1-induced EMT in GC. The mesenchymal phenotype is maintained via TGF-β1 expression in both tumor cells and immune cells in the tumor microenvironment, and is supported by CCR7, which drives lymph node metastasis and a poorer OS among patients strongly expressing CCR7.

Interestingly, we also showed that NF-κB signaling is involved in the crosstalk between CCR7 and TGF-β1-induced EMT in GC. NF-κB signaling was recently reported to trigger the progression of TNF-α-induced EMT in breast cancer by activating Twist [40]. It was also shown that NF-κB mediates TGF-β1-induced EMT and is predictive of tumor recurrence in patients with prostate cancer [41]. We have now verified, for the first time, that activation of the NF-κB signal can occur downstream of CCR7 and contribute to TGF-β1-induced EMT. These findings suggest evaluation of CCR7, TGF-β1 and NF-κB signaling in resected specimens after surgery may provide useful prognostic information in GC. Our data further suggest that the cross-talk between these signaling molecules represents a potential therapeutic target for preventing tumor metastasis.

## MATERIALS AND METHODS

### Cell lines

The SGC-7901, MGC80-3, AGS and N87 human gastric adenocarcinoma cell lines were obtained from Zhongshan Hospital, Shanghai Medical College. SGC-7901 was maintained in RPMI-1640 medium (Biowest, France) supplemented with 10% fetal bovine serum (FBS; Biowest, France). MGC80-3, AGS and N87 cells were maintained in Dulbecco's modified Eagle's medium (DMEM; Biowest, France) supplemented with 10% FBS.

### TGF-β1 treatment

Cells were seeded onto 60-mm dishes and allowed to attach, after which the medium was changed to RPMI or DMEM containing 1% FBS with or without TGF-β1 (2, 5, 10 or 20 ng/mL, PeproTech, USA) and the culture was continued for an additional 24 h before harvest.

### Western blotting

Whole-cell lysates was prepared by suspending cells in RIPA lysis buffer (Beyotime, China), after which protein concentrations were measured using a BCA protein assay kit (Beyotime, China). Equal amounts of denatured proteins were used for immunoblotting with primary antibodies. The western blotting procedures were carried out as previously described [42]. Sources of antibodies were detailed in the Supplementary Supporting Materials and Methods.

### Immunofluorescent staining

Immunofluorescent staining was performed as described previously [42]. Briefly, cells were seeded onto cover slips in 24-well plates and allowed to attach. The medium was then changed to RPMI or DMEM containing 1% FBS with or without TGF-β1, and the culture was continued. Representative images were obtained using a laser-scanning confocal microscope (magnification, 400× ; TCS-SP5; Leica Camera AG; Germany).

### Migration assays

Cell motility was assessed using wound-healing assays and transwell invasion assays. Details of these methods are provided in the Supplementary Supporting Materials and Methods.

### Patients and follow-up

This study was approved by the Zhongshan Hospital research ethics committee. The study participants were 133 patients with gastric carcinoma who underwent D2 resections between 1999 and 2005 at Zhongshan Hospital (Shanghai, China), as described previously [43]. The cancers were staged according to the TNM classification for gastric carcinoma (UICC). Details of the patient follow-up are provided in the Supplementary Supporting Materials and Methods. Ultimately, 11 patients were lost to follow-up so that data from only 122 patients were used for statistical analysis.

### Archived tissue for tissue microarrays

Archived tissues for tissue microarray (TMA) construction were collected from the study participants as previously described [43]. Details of the TMA construction are provided in the Supplementary Supporting Materials and Methods.

### Immunohistochemistry and evaluation of immunohistochemical variables

Tissue sections were immunostained using a two-step protocol (Novolink Polymer Detection System, Novocastra, Newcastle, UK) according to the manufacturer's instructions. Positively stained cells were scored on a scale of 0 to 3: 0 (< 20% cell staining), 1 (20–49% cell staining), 2 (50–74% cell staining) and 3 (75–100% cell staining). The data were analyzed using Leica QwinImage Processing and Analysis Application software (Wetzlar, Germany) by one student (F.W.) and one pathologist (L.Z.) who were blinded to all clinical information.

### Bioinformatic analysis

The network composed of CCR7 and its downstream genes was constructed using Exploratory Gene Association Networks (EGAN) software [29]. Pathway enrichment was based on the Kyoto Encyclopedia of Genes and Genomes (KEGG, http://www.genome.jp/kegg/) pathways database using DAVID Bioinformatics Resources 6.7 (http://david.abcc.ncifcrf.gov/home.jsp) [30].

### Statistical analysis

Actuarial OS rates were calculated using the Kaplan-Meier method and analyzed using the log-rank test. Univariate and multivariate analyses were based on the Cox proportional hazards regression model. For comparison of individual variables, χ^2^ tests and Fisher's exact tests were carried out as appropriate. Two-tailed *P* < 0.05 was considered significant. All analyses were performed using SPSS 20.0 software (SPSS, Chicago, IL).

## SUPPLEMENTARY MATERIALS AND METHODS


