# Supplementary material for: CCR7 enhances TGF-β1-induced epithelial-mesenchymal transition and is associated with lymph node metastasis and poor overall survival in gastric cancer
Source: Oncotarget. 2015 Jul 6;6(27):24348–60. doi: 10.18632/oncotarget.4484 (PMC4695190; doi:10.18632/oncotarget.4484)
Supplement: Supplementary file 1 [file oncotarget-06-24348-s001.pdf]

SUPPLEMENTARY MATERIALS AND METHODS

Primary antibodies used in western blot

Antibodies against CCR7(1:2000, Epitomics, USA), Fibronection (1:4000, Abcam, MA), E-cadherin (1:2000, Epitomics, USA), N-cadherin (1:6000, Epitomics, USA), Vimentin (1:3000, Epitomics, USA), MMP-9 ((1:500, Abcam, MA), β-catenin (1:2000, Abcam, MA), Snail (1:500, Abcam, MA), Twist (1:100, Abcam, MA), TGF-β1 (1:500, Abcam, MA), TNF-α(1:500, Abcam, MA), SMAD2 (1:500, BBI, China), NF-κB p65 (1:500, CST, MA), Phospho- NF-κB p65 (1:500, CST, MA) and loading control GAPDH (1:10000, KangChen Bio-tech Inc., China) and HRP conjugated second antibody (1:4000, KPL, USA). The visualization of the signals was by the ECL reagents (Thermo Fisher Scientific Inc., MA). Band intensities were qualified with a Gel-Pro analyzer (Media Cy-bernetics Inc., Silver Spring, MD).

Patients follow-up

Follow-up was completed on June 10, 2014. Follow-up procedures consisted of interim history, physical examination, tumor markers (CEA, CA199), abdominal ultrasonography and X-ray every 4–6 months according to the postoperative time. For patients with test results suggestive of recurrence, CT and MRI was used for corroborative evidence of relapse. The recurrences of gastric carcinoma had to be confirmed by cytology biopsy or surgery. OS was defined as the interval between surgery and death or between surgery and the last observation for surviving patients. The data were censored at the last follow-up for living patients.

Archived tissues for tissue microarray (TMA) construction

All cases were histologically verified by haematoxylin and eosin staining, and representative areas with small round lymphocyte infiltrate were remarked in the paraffin blocks, away from necrotic and haemorrhagic materials. Duplicate 1-mm diameter cylinders from two different areas, tumor center and nearest noncancerous margin (designated as intratumour and peritumour, respectively), that was, a total of four punches, were included in each case, together with different controls, to ensure reproducibility and homogenous staining of the slides (Shanghai Biochip Co., Ltd, Shanghai, China). Furthermore, three N1 regional lymph nodes which were the most adjacent to the tumor were also obtained from each case to make another tissue section. In short, each patient sample was constructed with four different tissue microarray blocks and 3 lymph nodes nearest to the tumor. Sections of 4-μm thickness were taken on 3-aminopropyltriethoxysilane-coated slides.

20 healthy gastric tissue samples were also supported by Shanghai Biochip Co., Ltd. Sections of 8-μm thickness were taken on 3-aminopropyltriethoxysilane-coated slides before used as control.

Migration and invasion assay

In the wound-healing assay for migration ability, cells were seeded into six-well plates. After cultured to 100% confluence, a wound with constant width was cut with a 200 μL micropipette tip in each well. Images of cell migration into the wounds were captured 12 hours with bright field microscopy and then analyzed with Image Pro Plus (USA). Independent *T*-test was conducted to determine the significance of the migratory effect.

In the transwell assay for the invasion ability, the lower chamber was added 500 μL 20% FBS medium and infiltrated the transwell inserts (8-μm, Corning, USA) for 2 h. After trypsinizing, the cells were aliquoted to four tubes which were mixed with or without TGF-β1 and mouse anti-human CCR7 neutralizing antibody (R&D Systems, USA). Each upper chamber was added 100 μL cell suspension. The plates were incubated at 37°C for 24 hours. After washing with 1 × PBS, the inserts were fixed in 4% PFA for 15 minutes. The cells in the upper portion of the filter membrane were removed by cotton swabs. Crystal violet was used to stain the cells left on the filter membrane for 10 minutes. The cells invaded onto the lower portion of the filter membrane were qualified under microscope with four random fields.
